# Heterozygous Mutations in DNA Repair Genes and Hereditary Breast Cancer: A Question of Power

**DOI:** 10.1371/journal.pgen.1003008

**Published:** 2012-09-27

**Authors:** Nathan A. Ellis, Kenneth Offit

**Affiliations:** 1 Department of Pediatrics and the Institute of Human Genetics, University of Illinois at Chicago, Chicago, Illinois, United States of America; 2 Department of Medicine, Memorial Sloan-Kettering Cancer Center, Program in Cancer Biology and Genetics, Sloan-Kettering Institute, New York, New York, United States of America; University of Washington, United States of America

The emerging technology of massively parallel DNA sequencing has had a major impact on progress in genomics and personalized medicine [1]. Most recently, DNA sequencing of whole exomes (complete coding regions of the human genome) has revealed the genetic basis of many previously-not-localized Mendelian traits [2]. In diseases where the underlying genetic basis is more dilute and complex, old challenges reappear in new clothes [1], [3].

Both the promise and the limitations of these new technologies have been evident in the untangling of the polygenic basis of susceptibility to human breast cancer. The identification of single-gene defects in cancer susceptibility syndromes in the 1990s provided a deterministic model of genetic susceptibility to cancer. Discovery and genetic analysis of the hereditary breast and ovarian cancer genes *BRCA1* and *BRCA2* offered a preview of personalized genomics, improving medical management of a common form of inherited human neoplasia [4], [5]. Supporting the idea that new breast cancer genes (often referred to collectively as *BRCA3*) could be identified, analysis of the genetic variance remaining after *BRCA1* and *BRCA2* mutations had been excluded suggested that most of the excess genetic risk was concentrated in a small percentage of persons [6]. Yet, genetic linkage studies provided little encouragement for the existence of *BRCA3* [7]. While genome-wide association studies (GWAS) uncovered new pathways in cancer biology, the GWAS results identified markers of very modest effect size [8]. The realization that a small proportion of excess genetic risk can be accounted for by common variants has resulted in a return to the study of multiplex breast cancer kindreds and the utilization of massively parallel DNA sequencing to uncover rare, disease-causing mutations in hereditary breast cancer.

The article by Thompson et al. published in this issue of *PLOS Genetics* [9], along with a similar article published recently in the *American Journal of Human Genetics* [10], provide a glimpse of the early applications of new sequencing technologies to the search for the “missing heritability” in hereditary breast cancer. In Thompson et al., the authors performed exome sequencing of multiple breast cancer cases from a small number of families (33 persons in 15 families) in whom *BRCA1* and *BRCA2* mutations had been excluded, and they focused on mutations that are predicted to ablate the function of the gene product, namely, mutations that cause premature termination of translation or that destroy splice-sites. After filtering out the overtly deleterious mutations that are polymorphic in the human population (under the assumption that the risk-causing variation in these breast cancer families should be rare), each sequenced individual harbored on average 35 overtly deleterious mutations. Thus, additional filtering criteria were needed to narrow the field: various strategies are possible, and here the authors focused on genes both hit by mutation in multiple individuals and participating in DNA repair through the homologous recombination pathway, a pathway that repairs double-strand breaks with high fidelity. Previous candidate-gene DNA sequencing studies have implicated homologous recombination in breast cancer susceptibility [11]–[16], and *BRCA1* and *BRCA2* themselves are players in this pathway [17]. Remarkably, two families carried overtly deleterious mutations in the Fanconi anemia (FA) gene *FANCC* and one family carried an overtly deleterious mutation in the Bloom's syndrome (BS) gene *BLM*.

FA and BS are rare, autosomal recessive conditions that are characterized by multiple developmental abnormalities (small size and congenital defects of the dermal, immune, skeletal, and reproductive systems), striking DNA repair defects and genomic instability in the somatic cells, and enormous predisposition to the development of various cancers (Figure 1) [18], [19]. Bi-allelic mutations in *FANCC* and *BLM* result in FA and BS, respectively, whereas in Thompson et al. heterozygous mutations in *FANCC* and *BLM* were identified in a few breast cancer families studied. The notion that heterozygous mutations in DNA repair genes might predispose carriers to incremental increases in cancer susceptibility is a long-standing and sometimes controversial hypothesis in cancer genetics that has increasingly gained traction. As noted above, heterozygous mutations in FA genes have been associated previously with increased breast cancer risk, and, conversely, bi-allelic mutations in breast cancer-associated genes *BRCA2*, *BRIP1*, *PALB2*, and *RAD51C* have been identified in persons with FA or FA–like syndromes [20]–[23]. Although the concept of increased cancer risk conferred by heterozygous mutations now seems unassailable, the evidence for specific associations between *FANCC* and *BLM* and breast cancer risk is not yet convincing [24]–[28]. The challenge of the “heterozygous-mutation” hypothesis is generally one of power. Because the allele frequency of *FANCC* and *BLM* mutations in most populations is very low (<0.001), large numbers of individuals are needed to test for differences in the allele frequency between cases and controls. Moreover, heterogeneity in the frequency of mutations across different populations could complicate interpretation of associations when populations are admixed and as investigators combine results from different populations to increase power. As an example of frequency heterogeneity, *FANCC* and *BLM* mutations are more frequent in Ashkenazi Jews (∼0.008), where a specific allele is present in most cases of FA and BS [29], [30].

**Figure 1:**
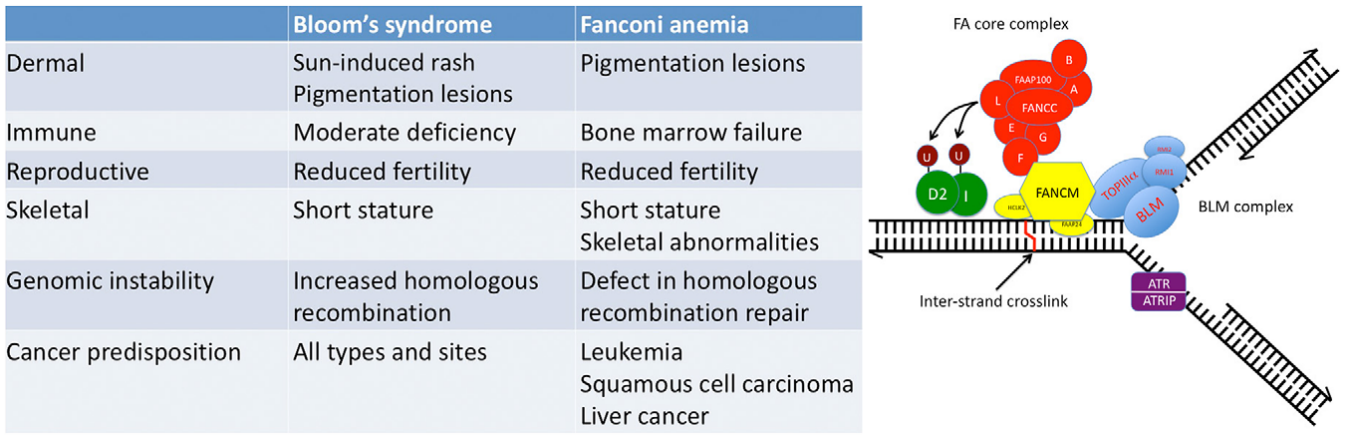
Fanconi anemia (FA) and Bloom's syndrome (BS) overlap at the clinical and molecular levels. Left panel: Comparison of the clinical and cellular features present in FA and BS. FA and BS have features that are distinct to each syndrome; however, there are broad similarities. Some individuals with mutations in *BLM* have been diagnosed with FA, exhibiting classical FA features. Right panel: Depiction of a super-complex that is formed at sites of repair of replication forks that have been impeded by an inter-strand crosslink. The super-complex consists of two complexes that form independently in the nucleus. One complex consists of the interacting proteins identified through FA mutations and the other complex consists of proteins that interact with BLM, the gene mutated in BS [33]. These two complexes are brought together by mutual interactions with the FA gene product FANCM [34]. Molecular interactions between FANCJ and BLM at stalled forks have also been described [35]. Downstream signaling effects (FAND2 ubiquitylation and ATR activation) are also depicted. Figure was redrawn from Deans and West [34].

Selecting cases with strong family history of cancer is an enrichment strategy that can reduce the numbers of cases needed to find associations [31]. In Thompson et al., the authors sequenced *FANCC* and *BLM*, and in total they found *FANCC* mutations in four probands out of 1,395 *BRCA*-negative hereditary breast cancer families from Australia and *BLM* mutations in two probands out of 438 such families. No mutation carriers were found in either gene in 464 controls. No overtly deleterious *FANC* or *BLM* mutations have been reported in 1,192 completely sequenced persons in the 1000 genomes data [32]. On the other hand, in the NHLBI GO Exome Sequencing Project (http://evs.gs.washington.edu/EVS/), three *FANCC* and four *BLM* mutation carriers had been identified in 3,510 exomes. Persons from the 1000 genomes and EVS do not constitute a good control group; thus the authors refrained from calculating *p*-values, 95% confidence intervals, and effect sizes. Going forward it will be important to compare consecutive breast cancer cases and matched controls drawn from the same population to provide robust data to calculate these important parameters; it may take tens of thousands of cases and controls to quantify them! In addition, we will need to combine studies of hereditary breast cancer cases to increase power and drive segregation analysis, to conduct larger case-control studies in the Ashkenazi Jewish population where the increased frequency of specific alleles increases the power of the study group, and to continue to quantify cancer risk in the relatives of persons with FA and BS.

Thus, rare alleles identified by sequencing of multiplex kindreds pose significant challenges for the estimation of effect sizes in cancer susceptibility. In the end, the critical questions that grip rare alleles are how much increased risk do they confer and do they account for the missing heritability? The resolution of these questions is of paramount importance to genetic epidemiologists studying human populations as well as to clinicians caring for families at risk for hereditary breast cancer.

## References

[1] Offit K (2011) Personalized medicine: new genomics, old lessons. Hum Genet 130: 3–14. 21706342 10.1007/s00439-011-1028-3 PMC3128266

[2] Bamshad MJ, Ng SB, Bigham AW, Tabor HK, Emond MJ, et al (2011) Exome sequencing as a tool for Mendelian disease gene discovery. Nat Rev Genet 12: 745–755. 21946919 10.1038/nrg3031

[3] Kiezun A, Garimella K, Do R, Stitziel NO, Neale BM, et al (2012) Exome sequencing and the genetic basis of complex traits. Nat Genet 44: 623–630. 22641211 10.1038/ng.2303 PMC3727622

[4] Robson M, Offit K (2007) Clinical Practice. Management of an inherited predisposition to breast cancer. N Engl J Med 357: 154–62. 17625127 10.1056/NEJMcp071286

[5] Kauff ND, Satagopan JM, Robson ME, Scheuer L, Hensley M, et al (2002) Risk-reducing salpingo-oophorectomy in women with a BRCA1 or BRCA2 mutation. N Engl J Med 346: 1609–1615. 12023992 10.1056/NEJMoa020119

[6] Antoniou AC, Pharoah PD, McMullan G, Day NE, Ponder BA, et al (2001) Evidence for further breast cancer susceptibility genes in addition to BRCA1 and BRCA2 in a population-based study. Genet Epidemiol 21: 1–18. 11443730 10.1002/gepi.1014

[7] Thompson D, Szabo CI, Mangion J, Oldenburg RA, Odefrey F, et al (2001) Evaluation of linkage of breast cancer to the putative BRCA3 locus on chromosome 13q21 in 128 multiple case families from the Breast Cancer Linkage Consortium. Proc Natl Acad Sci U S A 99: 827–831. 10.1073/pnas.012584499 PMC117390 11792833

[8] Stadler ZK, Thom P, Robson ME, Weitzel JN, Kauff ND, et al (2010) Genome-wide association studies of cancer. J Clin Oncol 28 27:4255–67. 20585100 10.1200/JCO.2009.25.7816 PMC2953976

[9] Thompson ER, Doyle MA, Ryland GL, Rowley SM, Choong DYH, et al (2012) Exome sequencing identifies rare deleterious mutations in DNA repair genes *FANCC* and *BLM* as potential breast cancer susceptibility alleles. PLoS Genet e1002894 doi:10.1371/journal.pgen.1002894. 23028338 10.1371/journal.pgen.1002894 PMC3459953

[10] Park DJ, Lesueur F, Nguyen-Dumont T, Pertesi M, Odefrey F, et al (2012) Rare mutations in XRCC2 increase the risk of breast cancer. Am J Hum Genet 90: 734–739. 22464251 10.1016/j.ajhg.2012.02.027 PMC3322233

[11] Meijers-Heijboer H, van den Ouweland A, Klijn J, Wasielewski M, de Snoo A, et al (2002) Low-penetrance susceptibility to breast cancer due to CHEK2(*)1100delC in noncarriers of BRCA1 or BRCA2 mutations. Nat Genet 31: 55–59. 11967536 10.1038/ng879

[12] Levran O, Attwooll C, Henry RT, Milton KL, Neveling K, et al (2005) The BRCA1-interacting helicase BRIP1 is deficient in Fanconi anemia. Nat Genet 37: 931–933. 16116424 10.1038/ng1624

[13] Renwick A, Thompson D, Seal S, Kelly P, Chagtai T, et al (2006) ATM mutations that cause ataxia-telangiectasia are breast cancer susceptibility alleles. Nat Genet 38: 873–875. 16832357 10.1038/ng1837

[14] Seal S, Thompson D, Renwick A, Elliott A, Kelly P, et al (2007) PALB2, which encodes a BRCA2-interacting protein, is a breast cancer susceptibility gene. Nat Genet 39: 165–167. 17200668 10.1038/ng1959 PMC2871593

[15] Meindl A, Hellebrand H, Wiek C, Erven V, Wappenschmidt B, et al (2010) Germline mutations in breast and ovarian cancer pedigrees establish RAD51C as a human cancer susceptibility gene. Nat Genet 42: 410–414. 20400964 10.1038/ng.569

[16] Loveday C, Turnbull C, Ramsay E, Hughes D, Ruark E, et al (2011) Germline mutations in RAD51D confer susceptibility to ovarian cancer. Nat Genet 43: 879–882. 21822267 10.1038/ng.893 PMC4845885

[17] D'Andrea AD (2010) Susceptibility pathways in Fanconi's anemia and breast cancer. N Engl J Med 362: 1909–1919. 20484397 10.1056/NEJMra0809889 PMC3069698

[18] Auerbach AD, Joenje H, Buchwald M (2011) Fanconi anemia. In: Valle D, Beaudet AL, Vogelstein B, Kinzler KW, Antonarkis SE, et al.. Scriver's online metabolic and molecular bases of inherited disease. New York: McGraw Hill. Chapter 31.

[19] German J, Ellis NA (2011) Bloom syndrome. In: Valle D, Beaudet AL, Vogelstein B, Kinzler KW, Antonarkis SE, et al.. Scriver's online metabolic and molecular bases of inherited disease. New York: McGraw Hill. Chapter 30.

[20] Howlett NG, Taniguchi T, Olson S, Cox B, Waisfisz Q, et al (2002) Biallelic inactivation of BRCA2 in Fanconi anemia. Science 297: 606–609. 12065746 10.1126/science.1073834

[21] Levitus M, Waisfisz Q, Godthelp BC, de Vries Y, Hussain S, et al (2005) The DNA helicase BRIP1 is defective in Fanconi anemia complementation group J. Nat Genet 37: 934–935. 16116423 10.1038/ng1625

[22] Reid S, Schindler D, Hanenberg H, Barker K, Hanks S, et al (2007) Biallelic mutations in PALB2 cause Fanconi anemia subtype FA-N and predispose to childhood cancer. Nat Genet 39: 162–164. 17200671 10.1038/ng1947

[23] Vaz F, Hanenberg H, Schuster B, Barker K, Wiek C, et al (2010) Mutation of the RAD51C gene in a Fanconi anemia-like disorder. Nat Genet 42: 406–409. 20400963 10.1038/ng.570

[24] Gruber SB, Ellis NA, Rennert G, Offit K, et al (2002) BLM heterozygosity and the risk of colorectal cancer. Science 297: 2013. 12242432 10.1126/science.1074399

[25] Cleary SP, Zhang W, Di Nicola N, Aronson M, Aube J, et al (2003) Heterozygosity for the BLM(Ash) mutation and cancer risk. Cancer Res 63: 1769–1771. 12702560

[26] Berwick M, Satagopan JM, Ben-Porat L, Carlson A, Mah K, et al (2007) Genetic heterogeneity among Fanconi anemia heterozygotes and risk of cancer. Cancer Res 67: 9591–9596. 17909071 10.1158/0008-5472.CAN-07-1501 PMC3622247

[27] Baris HN, Kedar I, Halpern GJ, Shohat T, Magal N, et al (2007) Prevalence of breast and colorectal cancer in Ashkenazi Jewish carriers of Fanconi anemia and Bloom syndrome. Isr Med Assoc J 9: 847–850. 18210922

[28] Sokolenko AP, Iyevleva AG, Preobrazhenskaya EV, Mitiushkina NV, Abysheva SN, et al (2012) High prevalence and breast cancer predisposing role of the BLM c.1642 C>T (Q548X) mutation in Russia. Int J Cancer 130: 2867–2873. 21815139 10.1002/ijc.26342

[29] Whitney MA, Saito H, Jakobs PM, Gibson RA, Moses RE, et al (1993) A common mutation in the FACC gene causes Fanconi anaemia in Ashkenazi Jews. Nat Genet 4: 202–205. 8348157 10.1038/ng0693-202

[30] Ellis NA, Ciocci S, Proytcheva M, Lennon D, Groden J, et al (1998) The Ashkenazic Jewish Bloom syndrome mutation blmAsh is present in non-Jewish Americans of Spanish ancestry. Am J Hum Genet 63: 1685–1693. 9837821 10.1086/302167 PMC1377640

[31] Houlston RS, Peto J (2004) The search for low-penetrance cancer susceptibility alleles. Oncogene 23: 6471–6476. 15322517 10.1038/sj.onc.1207951

[32] MacArthur DG, Balasubramanian S, Frankish A, Huang N, Morris J, et al A systematic survey of loss-of-function variants in human protein-coding genes. Science 335: 823–828. 22344438 10.1126/science.1215040 PMC3299548

[33] Meetei AR, Sechi S, Wallisch M, Yang D, Young MK, et al (2003) A multiprotein nuclear complex connects Fanconi anemia and Bloom syndrome. Mol Cell Biol 23: 3417–3426. 12724401 10.1128/MCB.23.10.3417-3426.2003 PMC164758

[34] Deans AJ, West SC (2009) FANCM connects the genome instability disorders Bloom's syndrome and Fanconi anemia. Mol Cell 36: 943–953. 20064461 10.1016/j.molcel.2009.12.006

[35] Suhasini AN, Rawtani NA, Wu Y, Sommers JA, Sharma S, et al (2011) Interaction between the helicases genetically linked to Fanconi anemia group J and Bloom's syndrome. EMBO J 30: 692–705. 21240188 10.1038/emboj.2010.362 PMC3041957

